# Temporal and regional shifts of crop species diversity in rainfed and irrigated cropland in Iran

**DOI:** 10.1371/journal.pone.0264702

**Published:** 2022-03-11

**Authors:** Leila Jafari, Sara Asadi, Ashkan Asgari

**Affiliations:** 1 Assistant Professor of Horticultural Science Department, Faculty of Agriculture and Natural Resources, University of Hormozgan, Bandar Abbas, Iran; 2 Research Group of Agroecology in Dryland Areas, University of Hormozgan, Bandar Abbas, Iran; 3 Faculty of Agriculture, Ferdowsi University of Mashhad, Mashhad, Iran; 4 Assistant Professor, Minab Higher Education Center, University of Hormozgan, Bandar Abbas, Iran; Soil and Water Resources Institute ELGO-DIMITRA, GREECE

## Abstract

Concerns about the negative effects of declining agricultural biodiversity due to modern agricultural practices and climatic constraints in various parts of the world, including Iran, on the sustainability of agricultural ecosystems are increasingly growing. However, the historical knowledge of temporal and spatial biodiversity is lacking. To determine the value and trend of crop diversity in Iran, we used biodiversity indices based on the area under rainfed and irrigated crops and total cropland area from 1991 to 2018. There were large fluctuations in the amount of cultivated area in the past 30 years, peaking around 2005 to 2007 with about 13.1 million cultivated hectares. However, no general trend in increase or decrease of total cultivated land was shown. The crop species diversity of irrigated cropland was higher than the rainfed and total cropland. The Shannon diversity index showed a constant trend with a negligible slope, but species richness was increased, which was related to the rise in the area of some crop species in recent years. The area of wheat and barley had a significant impact on crop diversity, so Shannon diversity index reduced with their dominance. Overall, this study revealed that the Iranian agricultural system relies on wheat and barley. We warn that by increasing the area of these crops and the prevalence of monoculture, the probability of damage from external factors such as sudden weather changes or the spread of diseases will increase, leading to instability and production risks in the future.

## Introduction

Producing food, feed, fiber and fuel for a growing population is a major challenge [1, 2] and climate change can have a negative impact on it, thereby affecting nutrition and human health and future food security [3, 4]. Consequences of climate change such as drought, flood and frost with varying distribution and intensity during the growth and reproductive phases of plant species (short-term impacts) lead to disruption of a part of the food chain and crop distribution per year and increase harvest losses [4, 5]. For example, climate change is estimated to have reduced wheat and corn yields by 4 to 5 percent over the past 30 years [5]. Under these circumstances, a reasonable and cost-effective approach may be to create flexibility in agricultural systems by increasing crop diversity [5]. To illustrate, when precipitation is limited, crop diversity in the agricultural ecosystem can act as a catalyst for agricultural production [6]. In fact, diverse agroecosystems with higher resilience to climate change [7] and different types of risks such as droughts, floods, frost, weed or pest infestation, input supply risks, price and yield risks, and other types of risks [8] will continue to produce nutritious and healthy food and provide ecosystem services [9–11]. This means that crop diversification increases the capacity of agroecosystems and provides protection against environmental variability as different crops respond differently to change [7]. As a consequence, crop diversification in agroecosystem causes an increase in efficiency and productivity of resources and reduces risks [12, 13].

Biodiversity is related to vital ecological processes such as nutrient and water cycles, pest management, and disease control in agroecosystems [9, 14], which help maintain quantity, quality, and reliability of ecosystem services [15, 16]. Recent investigations indicate that diversification of agricultural ecosystems provides many advantages such as increasing crop yield, carbon sequestration, weed and pest suppression, etc. [6, 17–22]. Also, crop diversity has a significant effect on the stability and sustainability of crop production [23–26]. However, it has been proven that the proportion of natural and semi-natural habitats and traditional management practices may play a more important role in determining the level of biodiversity, providing habitats for a variety of species (such as animal and insect species in an agricultural ecosystem) and associated ecosystem services [27, 28]. Therefore, determining guidelines and policies to preserve and increase biodiversity in agricultural environments is important given the importance of agricultural systems in maintaining biodiversity in the region [29]. In some areas, the guidelines and policies affect natural habitats and in other areas only agricultural lands [27]. For example, one of the traditional agricultural practices characterized by minimal tillage and preservation of natural and semi-natural areas is agroforestry, which creates potential hotspots for biodiversity [27, 30, 31]. Moreover, the most traditional form of agricultural activity prevalent in many Asian countries and some tropical regions is increasing crop diversity through intercropping [32–34]. Compared to monoculture, this method not only reduces diseases and pests, but also minimizes soil erosion in various cropping systems with more intensive and extensive soils [35, 36].

Various mechanisms lie behind the positive impacts of crop diversity on biodiversity and ecosystem services (such as provisioning, supporting and regulating) [37, 38]. Globally, increasing crop diversity positively impacts all disease and pest management subsectors, i.e., disease, pest, and weed control, and crop damage reduction. Increasing crop diversity changes weed dynamics by limiting available resources such as water, light and minerals for weed species [38, 39]. Increasing canopy complexity in diversified cropping systems not only provides shelter to natural enemies, but also reduces the spread of diseases and pests through physical barriers and microclimatic changes [40, 41]. In addition, crop diversity improves all indicators of soil quality, including soil chemistry and physics and soil carbon content [42, 43]. Although increasing plant diversity in natural grasslands leads to increased soil carbon storage, enzymatic activity and microbial biomass [44, 45], the percentage of similar effects in diverse cropping systems is still debated. It is possible that increasing soil function and diversity may have a positive effect on crop yield by increasing nutrient mobilization and microbial activity or reducing the accumulation of pathogens [46–49]. This is while present agricultural approaches may have reduced the biodiversity of agroecosystems [50]. In contrast to multiple cropping, agricultural intensification, mechanization, specialization and increase in field size are decreasing agricultural diversity at the landscape level [51, 52].

On the other hand, the current approach of agriculture, which is more influenced by economic incentives and agricultural policies (influencing farmers’ decisions through financial assistance) to produce several selected crops with the belief in more monocultural production rather than diversified agricultural systems, is one of the obstacles to advance the strategy of increasing the flexibility of agricultural systems [7, 53]. But this approach, under the pretext of increasing population rates and the food they need, has led to fundamental contradictions, such as changing allocation, distribution of food growth potential, declining biodiversity, global environmental changes in water and soil quality, changes in the means of production, land allocation to fewer landowners, changes in class structures and undermining human cultures [54, 55]. Ironically, intensive agriculture and monoculture, by changing the water cycle, increasing pollution, and participating in climate change, threatens all the very important and effective bases that allow the green revolution. Thus, these contradictions, along with climate change over the coming decades, in addition to threatening the sovereignty, security and knowledge of indigenous peoples and peasants, and the security of modern food-dependent cultures, lead to reduced food quality, food shortages, and rising prices [4, 54].

On a global scale, crop diversity has increased linearly in recent decades, and this increase has been linked to agricultural economies and global diets. In particular, structural adjustment programs and agricultural trade liberalization in the 1980s have led to the production and export of several selected crops or genotypes, which had a major impact on crop selection and management at regional or national levels, especially in developing countries [56]. In recent decades, patterns of crop diversity change are expected to vary in different regions due to climatic constraints for crop growth. For example, the cropping systems in Iran are based on wheat and rice. The rice-based system is limited to land near the Caspian Sea, while the wheat-based system is predominant in the other regions of Iran. The dominance of wheat and rice in these regions has caused a reduction in crop diversity [57].

But how has the diversity of crop species in Iran changed recently? Here, to answer this question, we used the data of the area under cultivation from 1991 to 2018 to assess the value and trend of crop diversity. It was hypothesized that (1) crop diversity has changed in different agricultural systems (rainfed and irrigated cropland) and in different regions; (2) the pattern of crop diversity has changed over time under the influence of environmental conditions, especially water availability.

## Materials and methods

### Study area

**Fig 1:**
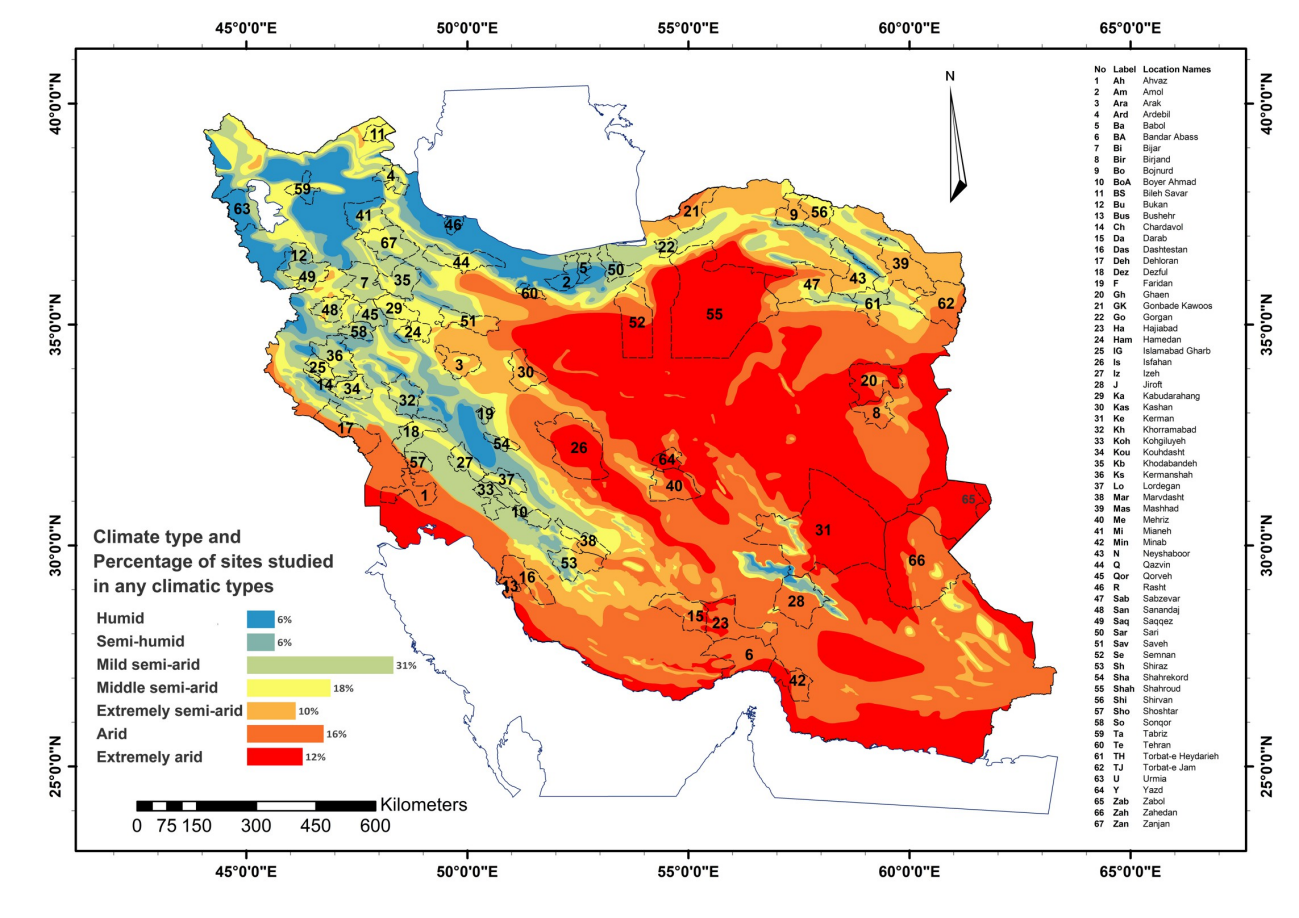
The geographical locations of the investigated sites (Dashed lines) based on the highest cropland area in Iran. The percentage of sites studied in any climatic types are shown in the legend of the figure. Dashed lines indicate Urban boundaries. Köppen-Geiger climate classification map obtained from Forest, Range and Watershed Managment organization (http://www.frw.ir). This maps was realised with ArcGIS 10.8 (http://www.esri.com/arcgis) using the georeferenced shapefiles obtained from the National Geographical Organization of Iran and National Cartographic Center (https://www.ncc.gov.ir/en/) at a scale of 1:11,000,000.

Iran is located in southwest of Asia that bounded by latitudes 25° to 45° N and longitudes 44° to 63.5° E (Fig 1). Two important mountain ranges of the country, including Zagros in the west and Alborz in the north, are the cause of non-uniformity of precipitation and humidity, which have led to different climatic conditions in this country. Humid and semi-humid climate prevails in the north of Iran and semi-arid climate prevails in the west and northwest of Iran. Extremely arid and arid climate mostly dominants over central, southern, and eastern Iran. For this investigation, 67 sites with different climate types were selected based on the highest cropland area and their importance in the agriculture of the country (at least two sites in each province). During the study period (1983 to 2018), 32.8%, 34.4% and 32.8% of these sites had an average cultivated area of less than 50, more than 50 and 100 thousand hectares, respectively. Details of the cropland area and the total area of the sites are shown in S1 Fig. The geographical location of the investigated sites, their climate conditions and the percentage of sites studied in any climatic types are shown in Fig 1.

### Index calculations

The most intuitive and basic measure to represent the biodiversity of a region is species richness (S), which is determined by counting the number of crop species in a habitat or region. Species richness is insensitive to abundance and the relative abundance distribution of crop species [58].

The Shannon-Wiener index (H’) is known as a measure of uncertainty based on the entropy of information. Uncertainty of the forecasts for a society that is dominated by one species (low diversity) is low; because, in sampling, the randomly selected species is likely to be the dominant species. Besides, uncertainty is high when species diversity in the society is high [59, 60]. H’ was calculated as follows [61]:

$$H^{\prime }=-\sum_{i=0}^{n}P_{i}\;\ln\;P_{i}$$ (1)

where pi (ni/N) is the proportion of the total crop area (N) that belongs to crop i(ni). The log was given in base 2 [62].

Another criterion that is broadly used in biodiversity measurement is the Simpson diversity index (1-D). This index indicates the probability that two randomly collected individuals from a community belong to different species [59, 63, 64]. The Simpson diversity index can be defined as follows:

$$1-D=1-\sum_{i=0}^{n}P_{i}^{2}$$ (2)

This equation is also used when using parameters such as canopy, biomass, production and area of crop. The value of the Simpson diversity index varies from zero to one. Higher values of the index (1) indicate higher diversity and vice versa [59, 64].

In addition, the Simpson evenness index was used to determine the value of evenness, which was calculated as follows [65].

$$E_{\frac{1}{D}}=\frac{\frac{1}{D}}{S}$$ (3)

where D is the Simpson index. The value of the Simpson evenness index varies from zero to one and is almost unaffected by rare species. The values close to zero and one represent lower and higher evenness, respectively.

### Spatial and statistical analyses

Three biodiversity indices, including Shannon diversity index (H’), Simpson diversity index (1-D) and Species richness index (S) were calculated for rainfed, irrigated and total cropland area of both types of cultivation in Iran between 1991 and 2018. For calculating H’ and E, species abundance values were considered equal to crop area. The ’vegan’ package in R software version 4.0.3 was used to calculate the biodiversity indices [66].

The interpolation of diversity indices was performed based on the inverse distance weighting (IDW) method using ArcMap 10.8 software. This method is more suitable than other interpolation options for applications to an abundance of data, species diversity and species richness, due to the limited interpolation values observed at the boundaries of observed maximum and minimum values [66, 67]. In fact, in methods like IDW, the original value of each station is not changed and other points are interpolated by averaging the values of the actual data in the neighborhood. In this method, the values of closer points have a greater effect on the predicted value than those of more distant points, which is called inverse distance. The default value for distance power is a two. The searching neighborhood was a circle, and the maximum and minimum numbers of neighbors to be included were 15 and 10, respectively. Cross-validation is a method of model validation used to assess how the result of a statistical analysis generalizes to an independent data set [68]. Cross-validation estimation is based on excluding a sample and validating the model with the remaining samples. The training dataset is used to build the predictive model, while the validation dataset is used to validate the model. In this study, the data were randomly divided into two groups of training and validation with a ratio of 80 to 20% using the Geostatistical Analyst tool in ArcGIS. The accuracy of interpolated points predicted based on the IDW algorithm with respect to the percentage of the actual data was determined by the root-mean-square error (RMSE).

Besides, the trend of H’, E and S during the last three decades and the relationship between these indices were investigated using polynomial and linear regression. Also, to determine a significant trend in H’, E and S, non-parametric Mann–Kendall test were applied. The Mann-Kendall test (MK) [69, 70] statistically evaluates if there is an upward or downward trend in the variable over time. A monotonic downward (upward) trend means that the variable constantly decreases (increases) over time, but the trend may or may not be linear. For more detailed information about the MK test, refer to Bannayan et al. [2] All data analyzes were performed in R using the R-packages ggplot2 [71] and Kendall [72].

## Results

### Fluctuations in the area under cultivation

**Fig 2:**
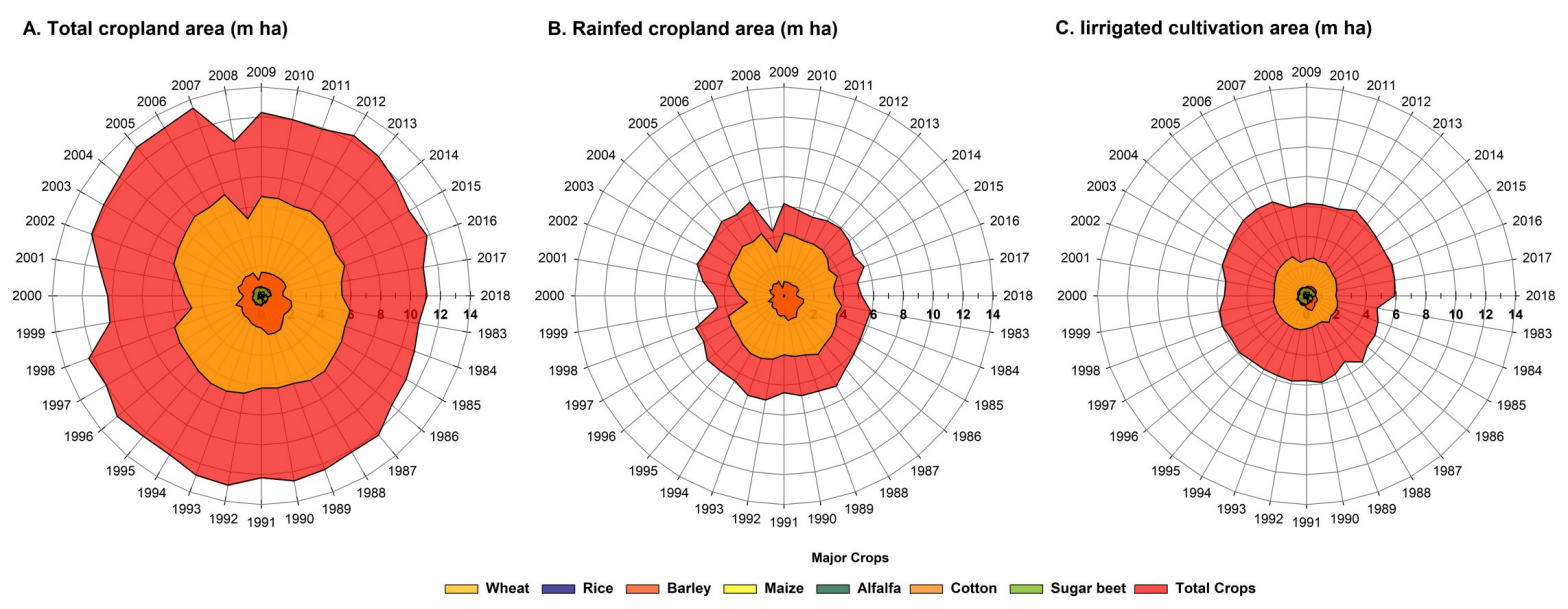
Area of 7 major crops and other crops and vegetables in total (A), rainfed (B) and irrigated (C) cropland in Iran between 1983 and 2018.

The results of this study showed that the total cropland area was almost constant trend with a negligible slope (with a slope of -0.00002 year-1) during the last three decades. However, the highest area under cultivation of about 13.1 million hectares during this period was in the years 2005 to 2007 (Fig 2A). Wheat was the predominant crop during this period, with the area under cultivation of more than 6 million ha in most years, except for two periods. The first and second negative peaks occurred from 1999 to 2001 and in 2008, which was very evident in the total cropland area. The predominant crop was followed by barley, alfalfa and rice. Until 1990, the growth rate for barley area was 0.101 million ha year^-1^, after which it declined to 0.164 million ha year^-1^. Although the growth rate for alfalfa and rice was 0.004 and 0.003 million ha year^-1^, respectively, their area was not significant compared to the area of wheat and barley (Fig 2A).

Two crops, wheat and barley, accounted for most of the area under rainfed cropping (Fig 2B). In the rainfed cropland area, the trend of changes in total crops and wheat (Fig 2B) was similar to the total cultivated area (Fig 2A), which shows the high impact of fluctuations of rainfed wheat area on the total cropland area.

In the irrigated cropland area, wheat was the predominant crop followed by barley, alfalfa and rice (Fig 2C). The area under barley was 0.69 million ha in most years, except one period. The positive peak occurred from 1987 to 1991. The growth rate of alfalfa and rice was 0.005 and 0.003 million ha year^-1^, respectively. The irrigated cropland area was significantly increasing (r = 0.72** and slope = 0.03) during the last three decades (Fig 2C).

### Shannon diversity index

**Fig 3:**
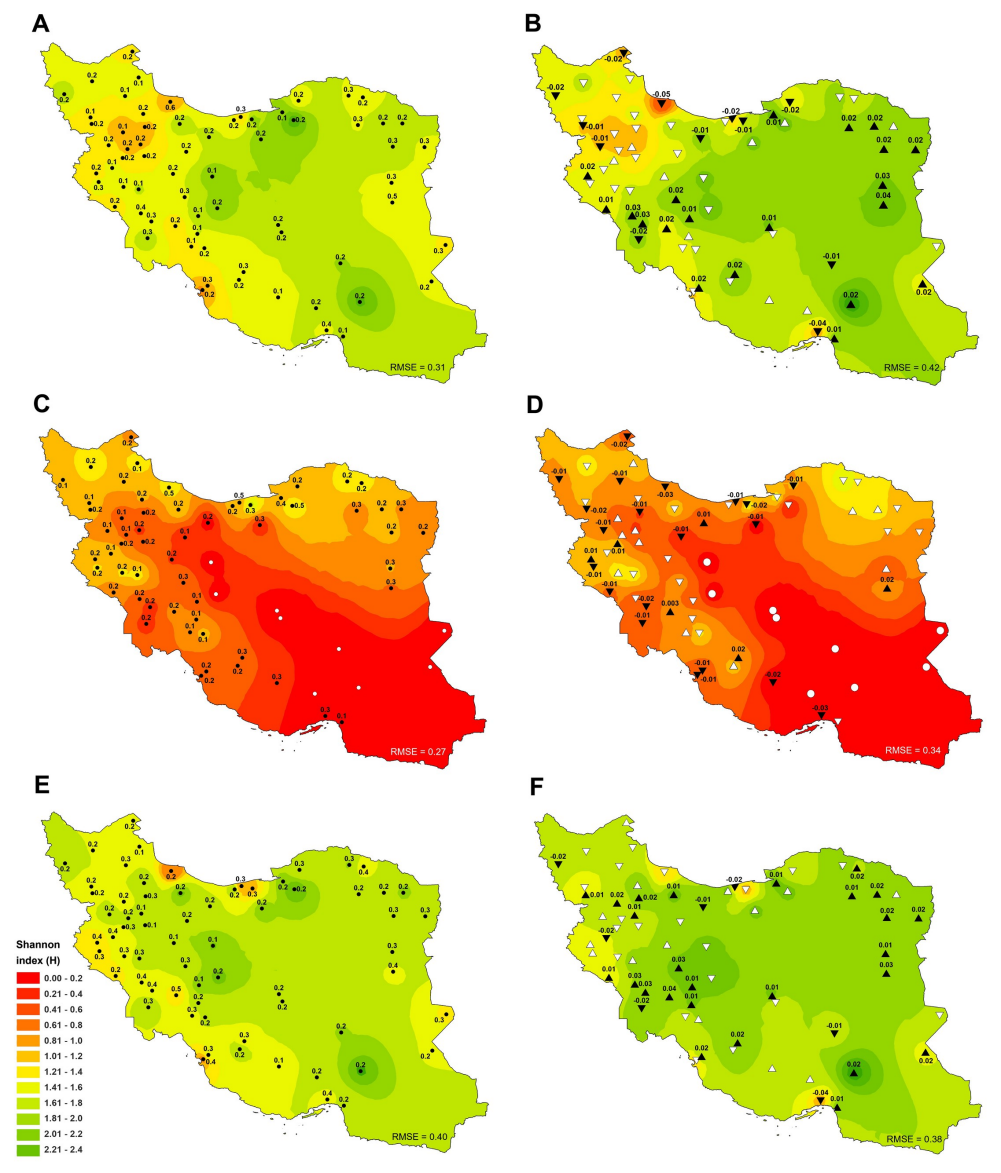
Spatial distribution of Shannon diversity index (H’) trends and tendencies in total (A, B), rainfed (C, D) and irrigated cropland (E, F) in Iran between 1991 and 2018. Significant positive and negative trends are shown by (▲) and (▼) and insignificant upward and downward trends are depicted by (△) and (▽), respectively. White circle (○) also indicates the lack of rainfed cultivation in the site. Besides, the numbers next to the triangle and circle indicate the average rate of change (slope) (B, D and F) and standard deviation (STDV) (A, C and E) in the variable, respectively. Complete time series are shown in S2 Fig. Also, the root-mean-square error (RMSE) of the interpolated values based on the inverse distance weighting method (IDW) to actual data values was specified at the bottom of each figure. Figures were realised with ArcGIS 10.8 (**http://www.esri.com/arcgis**) using the shapefile of the map of Iran obtained from NCC (**https://www.ncc.gov.ir/en/**) and Kendall R-package.

The average Shannon diversity index of all sites (irrigated and rainfed cropland) was 1.32 during 1991–2018 in the study area, with the highest and lowest values observed in Shahroud (2.19±0.2) and Bijar (0.5±0.1), respectively (Fig 3A and 3B). The positive and negative trends of Shannon index were significant (p ≤ 0.05) in approximately 18% and 30% of the sites, respectively (Fig 3B). The highest increase and decrease of Shannon index slope were achieved in Birjand (0.04) and Rasht (-0.05), respectively. The Shannon index ranged from 0.14 to 2.34 in 2018 with an average of 1.42, which was an improvement over the long-term average (Fig 3A and 3B).

The Shannon diversity index value for the country’s rainfed cropland ranged between 0 and 1.54 with an average of 0.61 during 1991–2018, and the highest Shannon index was for Sari (1.54±0.3). In nine sites, the Shannon index was zero due to the lack of cultivation of rainfed crops (Fig 3D). Also, the detected trend of Shannon index was negative in 59.7% of the sites (significant at 29.9% of them) and positive in 26.9% of the sites (significant at 9% of them) (Fig 3D). The low percentage of sites with a positive trend indicates the declining situation of crop diversity in rainfed cropland. On the other hand, Shannon index value was between zero and 1.39 with an average of 0.6 in 2018, indicating a decrease in crop diversity compared to the long-term average (Fig 3C). The highest Shannon index was recorded for Bojnourd (1.39±0.2) in 2018, followed by Sari (1.27±0.3). All the 14 sites with Shannon diversity index equal to zero (due to lack of rainfed cultivation) were located in the south, southeast and center parts of the country (Fig 3C and 3D).

The long-term average of Shannon index for irrigated cropland was 1.4. The highest and lowest crop diversity was observed in Shahroud (2.15±0.2) and Babol (0.12±0.3) during 1991–2018, indicating a better situation under irrigated conditions than under rainfed conditions (Fig 3F). Also, the trend of crop diversity was positive in 61.2% of the sites (significant at 35.8% of them) and negative in 38.8% of the sites (significant at 10.4% of them). The highest increasing and decreasing trend was related to Izeh (with a slope of 0.04 year^-1^) and Bandar Abbas (with a slope of -0.04 year^-1^), respectively (Fig 3F). In 2018, the highest and lowest values of Shannon index were observed in Jiroft (2.34±0.2) and Rasht (0.64±0.2), respectively (Fig 3E). The average Shannon index in 2018 (1.57) was increased by about 11.35% compared to the long-term average (Fig 3E and 3F).

### Simpson diversity index

**Fig 4:**
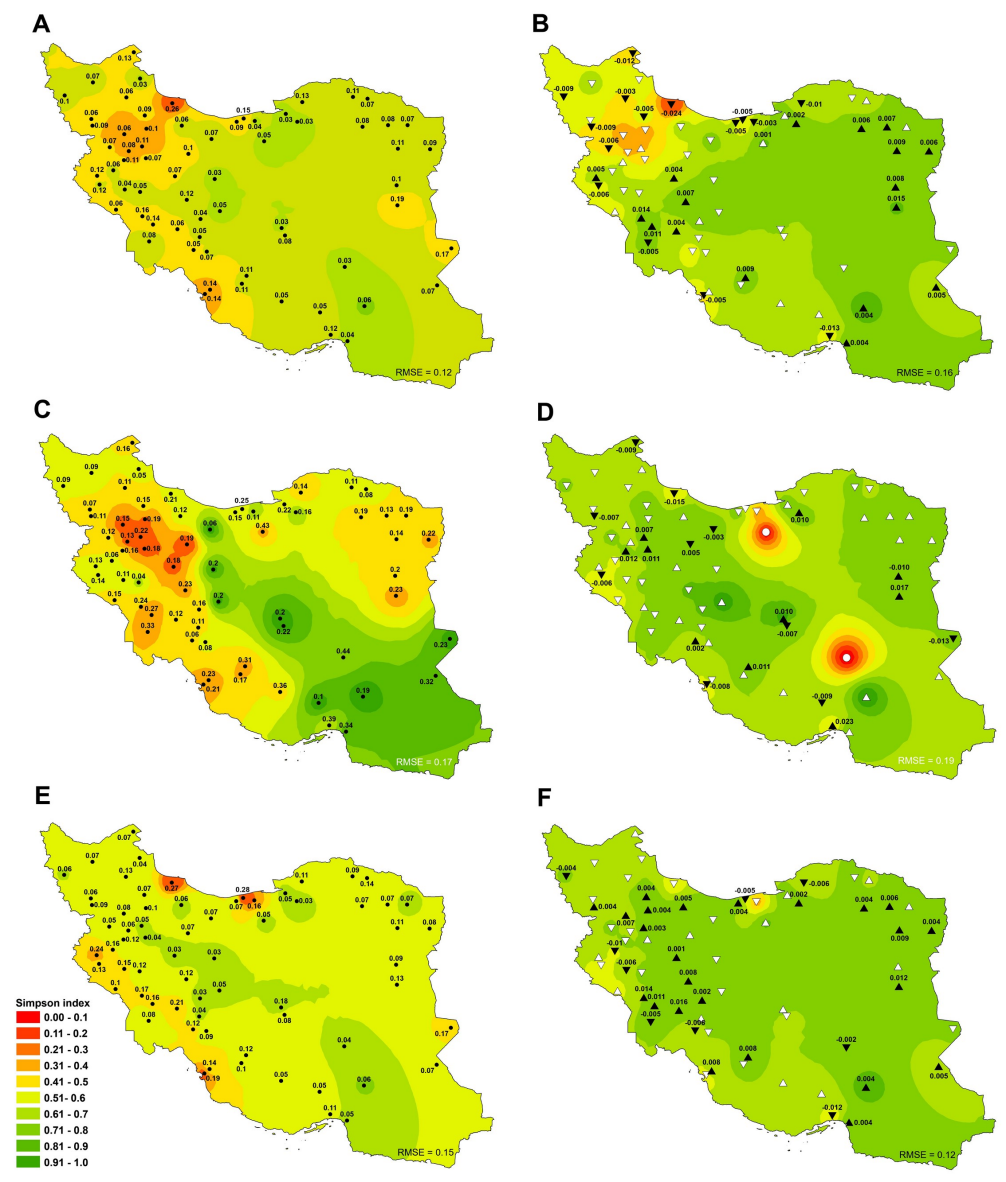
Spatial distribution of Simpson diversity index trends and tendencies in total (A, B), rainfed (C, D) and irrigated cropland (E, F) in Iran between 1991 and 2018. Significant positive and negative trends are shown by (▲) and (▼) and insignificant upward and downward trends are depicted by (△) and (▽), respectively. White circle (○) also indicates the lack of rainfed cultivation in the site. Besides, the numbers next to the triangle and circle indicate the average rate of change (slope) (B, D and F) and standard deviation (STDV) (A, C and E) in the variable, respectively. Complete time series are shown in S3 Fig. Also, the root-mean-square error (RMSE) of the interpolated values based on the inverse distance weighting method (IDW) to actual data values was specified at the bottom of each figure. Figures were realised with ArcGIS 10.8 (**http://www.esri.com/arcgis**) using the shapefile of the map of Iran obtained from NCC (**https://www.ncc.gov.ir/en/**) and Kendall R-package.

The average Simpson diversity index of all sites (irrigated and rainfed cropland) ranged from 0.21 to 0.83 (Country average: 0.6) (Fig 4A). The highest and lowest Simpson index were observed in Jiroft (0.83±0.06) and Bijar (0.21±0.06), respectively. This result shows that the suitable situation of Jiroft in terms of diversity of the crop species. It should be noted that the slope of changes in Simpson index was negligible during the study period (Fig 4B). The results for 2018 showed that the values of Simpson index were between 0.05 and 0.88 (country average: 0.63). The highest and lowest values of this index were also observed in Jiroft and Rasht (Fig 4B).

In rainfed cropland, the average Simpson index was 0.53 (Fig 4D) and the highest and lowest values of this index were observed in Hajiabad (0.97) and Saveh (0.12). During the same period, the Simpson index trend in 44.8 (significant at 14.9% of them) and 52.2% (significant at 14.9% of them) of the sites was increasing and decreasing, respectively, which indicates a decrease in crop species diversity (area cultivation). The highest increasing slope of Simpson index was obtained in Bandar Abbas (0.023) and the highest decreasing slope was obtained in Rasht (-0.015) (Fig 4D). On the other hand, the average, maximum and minimum of Simpson index in 2018 were equal to 0.68, 1 (Isfahan and Yazd) and zero (Semnan and Kerman), respectively, indicating an increase in Simpson index compared to the long-term average (Fig 4C and 4D).

In irrigated cropland, the Simpson index was 0.64 (Fig 4F). The highest and lowest values of this index were obtained from Jiroft (0.83) and Babol (0.13) during the same period, which respectively showed the similar area under cultivation of crop species in Jiroft and the dominance of some crop species such as rice in Babol. The trend of Simpson changes was such that 35.8% and 13.4% of the sites had a significant increase and decrease, respectively, which indicates an increase in diversity of cultivation area (Fig 4F). In 2018, Simpson index values were ranged from 0.3–0.88 with an average value of 0.7, indicated an increase compared to the long-term average; in other words, some crop species’ dominance was reduced (Fig 4E and 4F). The highest and lowest values were obtained from Jiroft (0.88±0.06) and Sari (0.3±0.16), respectively (Fig 4E).

#### Simpson evenness index

**Fig 5:**
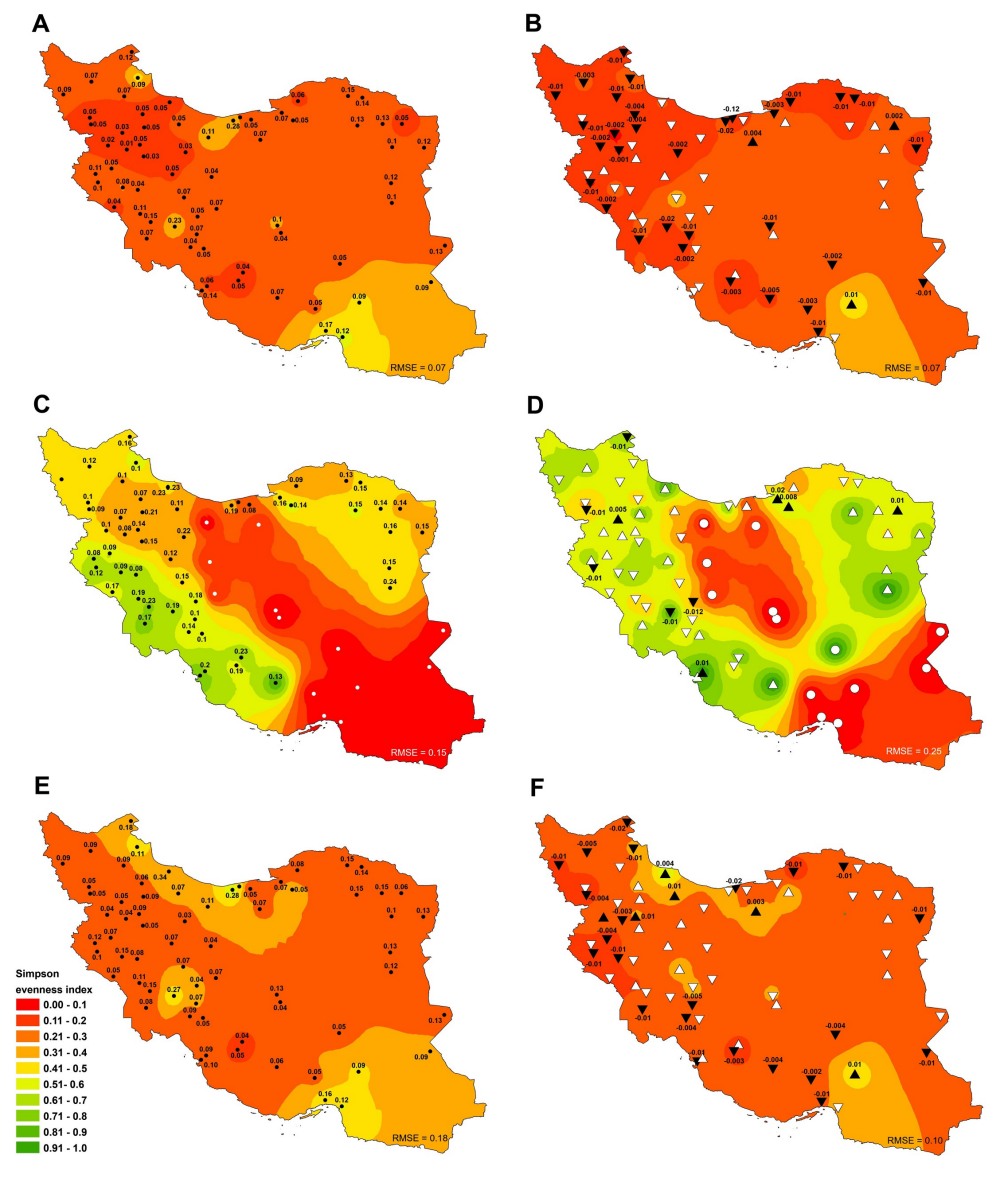
Spatial distribution of Simpson evenness index trends and tendencies in total (A, B), rainfed (C, D) and irrigated cropland (E, F) Iran between 1991 and 2018. Significant positive and negative trends are shown by (▲) and (▼) and insignificant upward and downward trends are depicted by (△) and (▽), respectively. White circle (○) also indicates the lack of rainfed cultivation in the site. Besides, the numbers next to the triangle and circle indicate the average rate of change (slope) (B, D and F) and standard deviation (STDV) (A, C and E) in the variable, respectively. Complete time series are shown in S4 Fig. Also, the root-mean-square error (RMSE) of the interpolated values based on the inverse distance weighting method (IDW) to actual data values was specified at the bottom of each figure. Figures were realised with ArcGIS 10.8 (**http://www.esri.com/arcgis**) using the shapefile of the map of Iran obtained from NCC (**https://www.ncc.gov.ir/en/**) and Kendall R-package.

The average Simpson evenness index of all sites (irrigated and rainfed cropland) ranged from 0.10 to 0.53 (country average: 0.24) during 1991–2018 (Fig 5A). The Simpson evenness index shows the evenness of the area under cultivation of the crop species. The lowest and highest Simpson evenness index, which indicates non-uniformity and uniformity of the area under cultivation of the crop species, were observed in Qorveh (0.10±0.09) and Minab (0.54±0.12). This result shows the suitable situation of Minab in terms of evenness of the crop species (Fig 5A). It should be noted that the slope of changes in Simpson evenness index was negligible during the study period (Fig 5B). The results for 2018 showed that the values of Simpson evenness index were between 0.08 and 0.48 (country average: 0.20). Also, the detected trend of Simpson evenness was significantly negative and positive for 47.8% and 4.5% of the sites, respectively (Fig 5B).

The value of Simpson evenness index for the country’s rainfed cropland was in the range of 0.24–0.89 with an average of 0.50 during 1991–2018 (Fig 5C). The Simpson evenness index for 13 sites was zero due to the lack of rainfed cropland in the south, southeast and center and parts of the north of the country (Fig 5C and 5D). The highest evenness was observed in Darab (0.89±0.07) during 1991–2018 and in Birjand, Dashtestan and Darab (one) in 2018. On the other hand, the value of Simpson evenness index was between 0.16 and one with an average of 0.6 in 2018, which indicates an increase in evenness compared to the long-term average (Fig 5C and 5D). The Simpson evenness index trend in 48.1 (significant at 9.3% of them) and 51.9% (significant at 9.3% of them) of the sites was decreasing and increasing (Fig 5D). The highest increasing slope of Simpson evenness was obtained in Gorgan (0.02) and the highest decreasing slope was obtained in Izeh, Shahrekord, Dezful, Shoshtar and Bilesavar (-0.01) (Fig 5D).

The long-term average of Simpson evenness index for irrigated cropland was 0.29. The highest evenness was found for Amol (0.61±0.21) followed by Izeh (0.56±0.27) during 1991–2018, which indicates more evenness under irrigated conditions than under rainfed conditions (Fig 5E). Also, the trend of Simpson evenness was negative in 70.1% of sites (significant at 34.3% of them) and positive in 29.9% of sites (significant at 9% of them), which indicates a decrease in evenness of cropland area (Fig 5F). The highest decreasing trend was related to Babol and Bilesavar (with a slope of -0.02 year^-1^) and the highest increasing trend was related to Kabudarahang, Qazvin and Jiroft (with a slope of 0.01 year^-1^) (Fig 5F). In 2018, the lowest and highest values of Simpson evenness index were observed in Sari (0.11±0.04) and Rasht (0.61±0.3), respectively (Fig 5F). The average of Simpson evenness index in 2018 (0.25) had decreased by about 13.8% compared to the long-term average (Fig 5E and 5F).

#### Species richness

**Fig 6:**
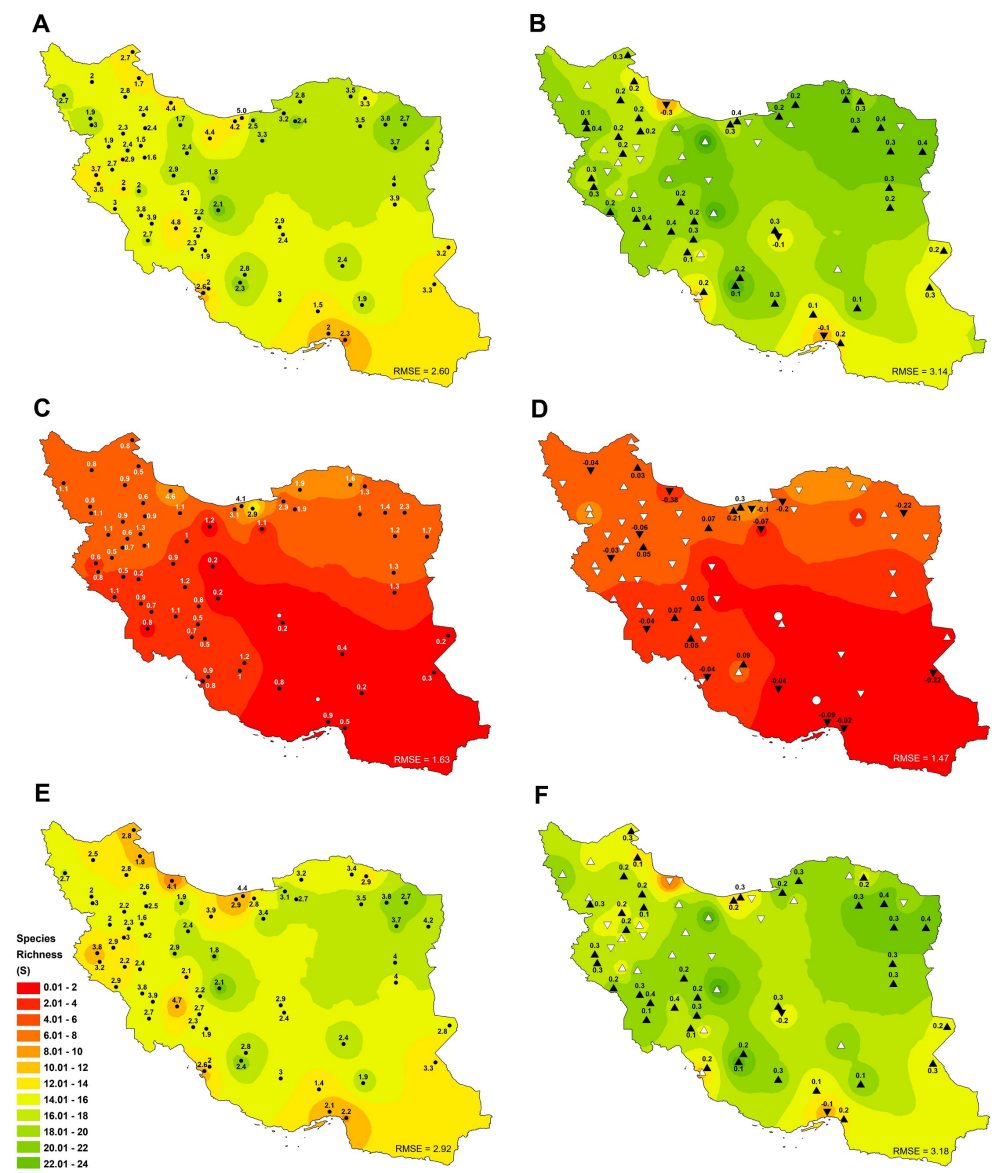
Spatial distribution of Species richness index (S) trends and tendencies in total (A, B), rainfed (C, D) and irrigated cropland (E, F) in Iran between 1991 and 2018. Significant positive and negative trends are shown by (▲) and (▼) and insignificant upward and downward trends are depicted by (△) and (▽), respectively. White circle (○) also indicates the lack of rainfed cultivation in the site. Besides, the numbers next to the triangle and circle indicate the average rate of change (slope) (B, D and F) and standard deviation (STDV) (A, C and E) in the variable, respectively. Complete time series are shown in S5 Fig. Also, the root-mean-square error (RMSE) of the interpolated values based on the inverse distance weighting method (IDW) to actual data values was specified at the bottom of each figure. Figures were realised with ArcGIS 10.8 (**http://www.esri.com/arcgis**) using the shapefile of the map of Iran obtained from NCC (**https://www.ncc.gov.ir/en/**) and Kendall R-package.

Investigation of species richness index in total cropland area of Iran (rainfed and irrigated cropland) showed that the average species richness was about 13.2 species (Fig 6B). The highest number of crop species was observed in Isfahan (19.6±2.1), which is affected by the number of crop species cultivated under irrigated (Fig 6B and 6F). In 2018, the species richness was increased by about 22.7% compared to the long-term average, which was due to the increasing number of crop species. Also, an investigation of the species richness index showed that 85.1% of sites had an increasing trend (significant at 59.7% of them), which indicates an increase in the number of crop species (Fig 6A and 6B). Moreover, there was a significant difference (p < 0.01) between the average species richness in irrigated (12.5) and rainfed (3.7) cropland (Fig 6D and 6F). In 2018, the number of irrigated and rainfed crop species was 15.3 and 3.8, respectively, indicating that species richness in irrigated condition was more suitable situation (Fig 6C–6F).

### Correlation of indices

**Fig 7:**
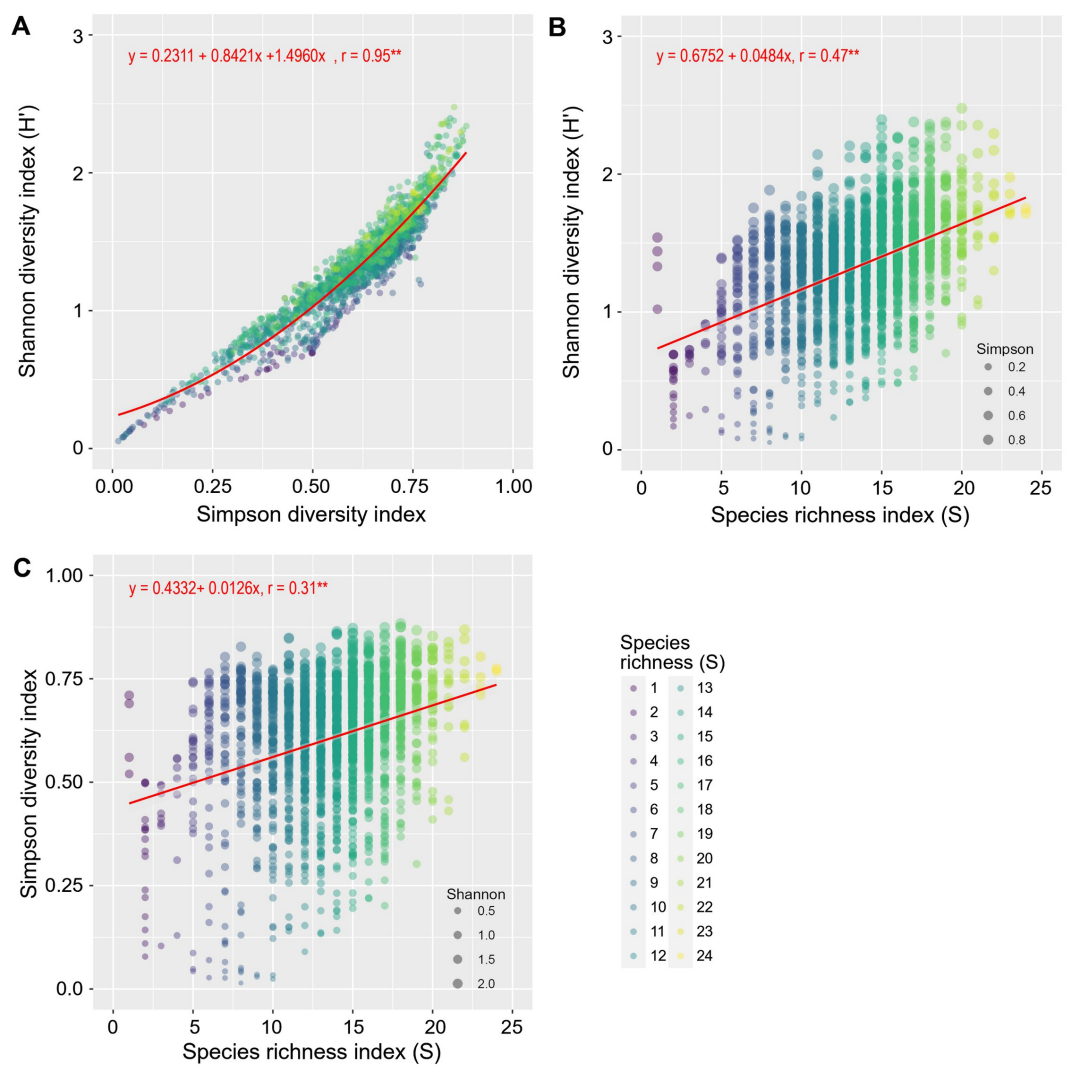
The relationship among Shannon diversity index (H’) (A), Simpson diversity index (B) and Species richness index (S) (C) in Iran between 1991 and 2018. The solid line is the polynomial (A) and linear (B, C) regression fit to the points (n = 1748). Significant trends (p ≤ 0.01) shown by **. Created using ggplot2 R-package.

The relationship among Shannon, Simpson and species richness indices was plotted with various regression equations in Fig 7. Each point in each figure represents the value of index for each site and year. There was a significant polynomial regression (r = 0.95**) between the Shannon and Simpson indices indicating that by increasing each index, the value of the other indices were also increased (Fig 7A). The Shannon index increased with increasing species richness. Since the sites with higher species richness were above the regression line, it can be concluded that increasing the species richness along with the Simpson index leads to increasing Shannon index with a greater slope compared to lower species richness (Fig 7A).

As shown in Fig 7B, high species richness does not necessarily lead to an increase in Shannon index. The pattern of point distribution in Fig 7B is almost similar to the shape of an almond. Sites with a lower Simpson value (smaller circles) are located on the almond’s lower edge. So, increasing species richness without increasing evenness does not increase Shannon index. Also, the lowest range of changes in Shannon index was obtained under the condition of increasing species richness (the end of the almond). If high species richness coincides with species evenness, it can increase Shannon index. Shannon index also had a positive and significant correlation (r = 0.47**) with species richness (Fig 7B). The results of the relationship between Simpson index and species richness (r = 0.31**) showed that by increasing the number of crop species, Simpson index was somewhat increased, but Simpson index actually indicates the evenness of crop species in terms of cultivated area and is more affected by the area under cultivation of species (Fig 7C).

### Correlation between the diversity index and the area under cultivation of wheat and barley

**Fig 8:**
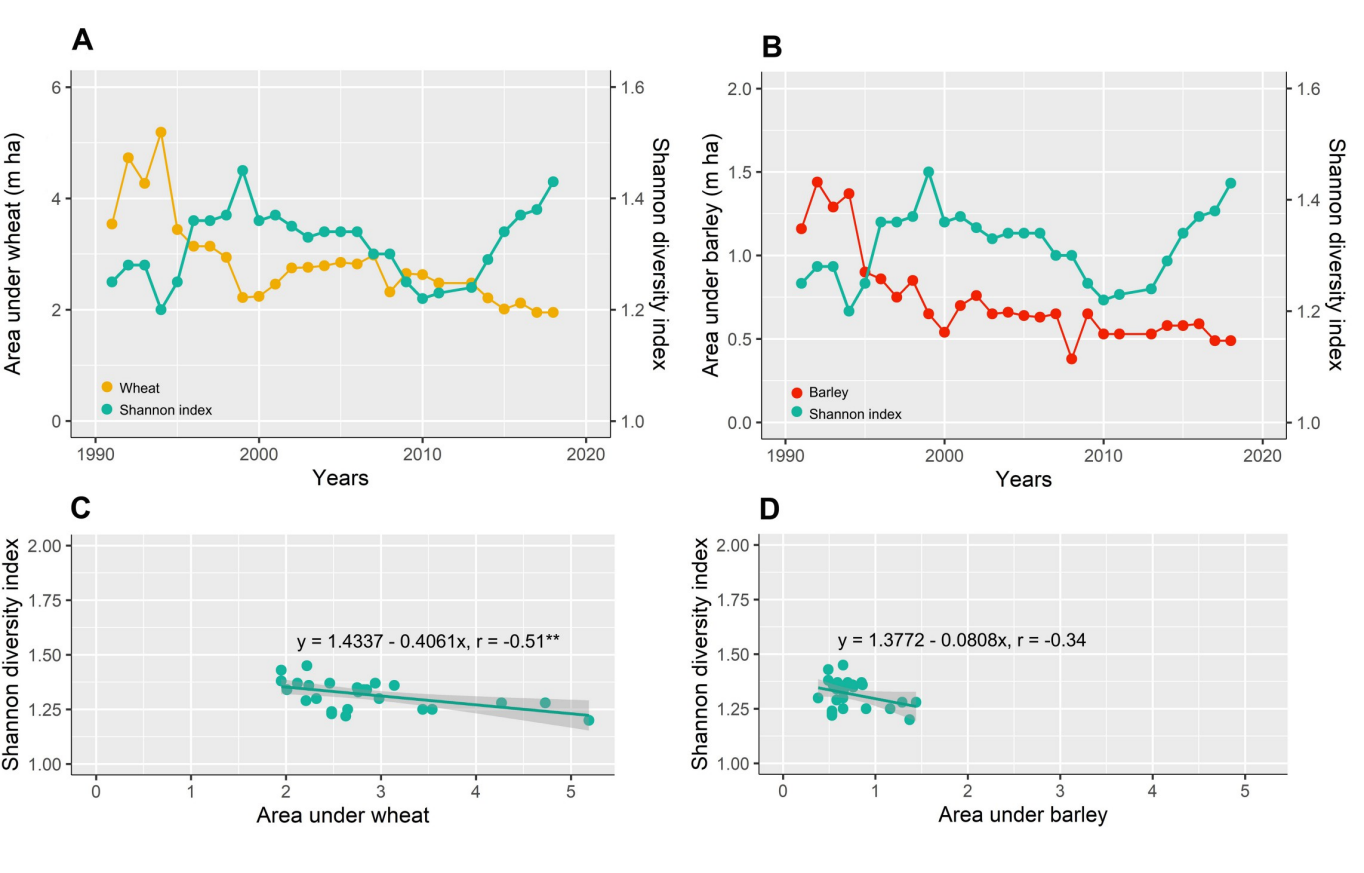
The trend of Shannon diversity index (H’) (A, B) and area under wheat (A) and barley (B) cultivation in Iran between 1991 and 2018. The relationship between Shannon diversity index (H’) and area under wheat (C) and barley (D) cultivation in Iran between 1991 and 2018. The solid line is the linear regression fit to the blue points (n = 28). Significant trends (p ≤ 0.01) shown by **. Created using ggplot2 R-package.

There was an overall negative relationship between Shannon index and wheat and barley cultivation (Fig 8). The relationship between wheat area and Shannon index was significantly decreasing nationwide (r = 0.51** and slope = -0.41) (Fig 8C). Also, there was also no significant decrease between barley area and Shannon index (r = 0.34 and slope = -0.08) (Fig 8D). Given that the Shannon index is calculated based on the number and cultivation area of crop species, as long as the species has an equal cultivation area, the Shannon index increases. Therefore, by increasing the area of wheat and barley due to the dominance, crop diversity was reduced (Fig 8). Investigation of the area of the dominant crops in the period showed that crop diversity indices were greatly affected by the area of wheat and barley.

**Fig 9:**
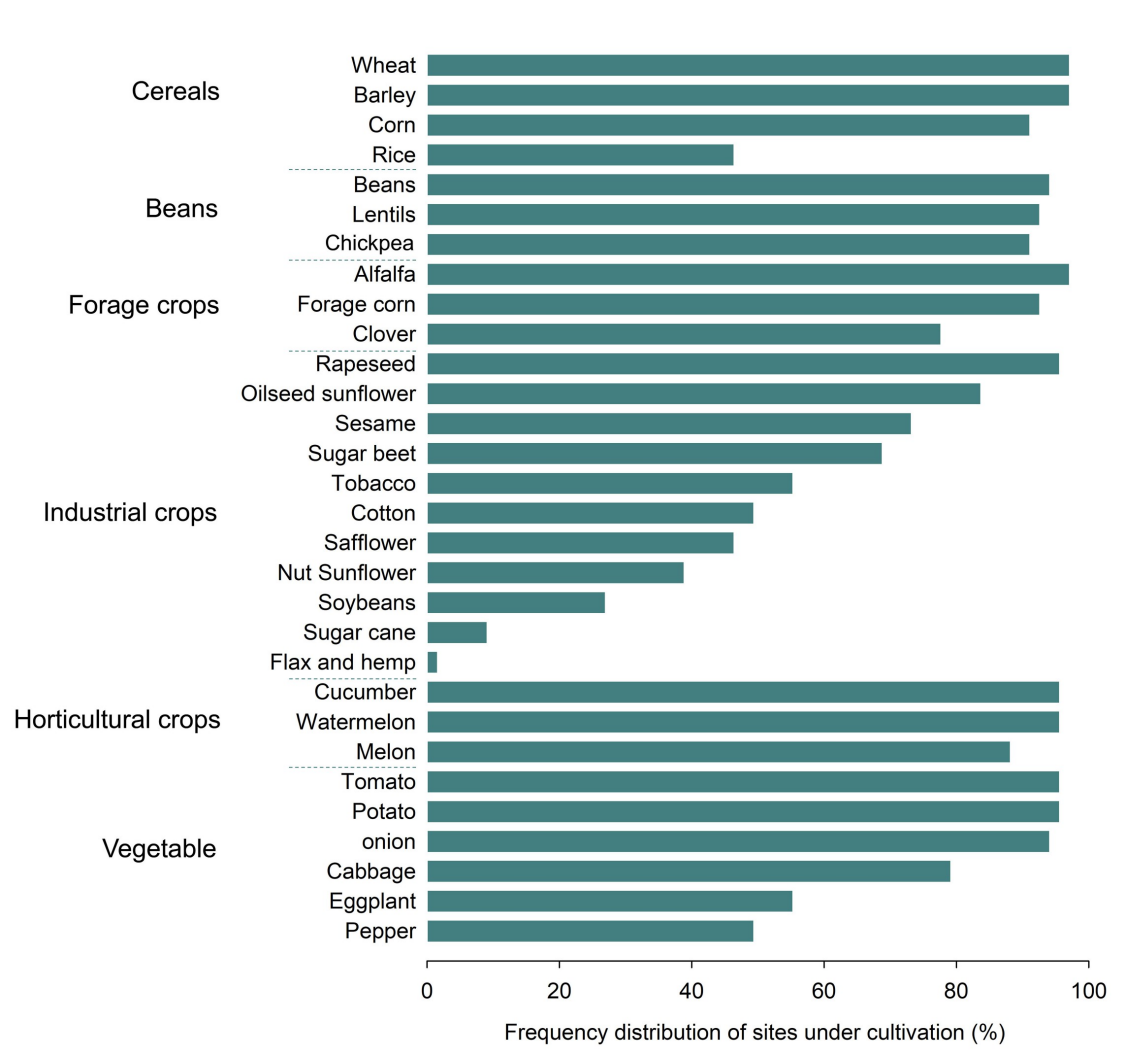
List of crops and the frequency distribution of sites under their cultivation in Iran. Created using ggplot2 R-package.

Investigation of crop species showed that some crop species such as wheat, barley, alfalfa, beans, potato, rapeseed, corn, lentils, onion, Chickpea, cucumber, tomato and watermelon had been cultivated in more than 90% of the study areas. Other crops such as sugarcane, flax and hemp were present in less than 10% of the study areas. Flax and hemp were present only in Sari (Fig 9).

### Changes in diversity indices as a result of different climates and cultivation patterns

**Fig 10:**
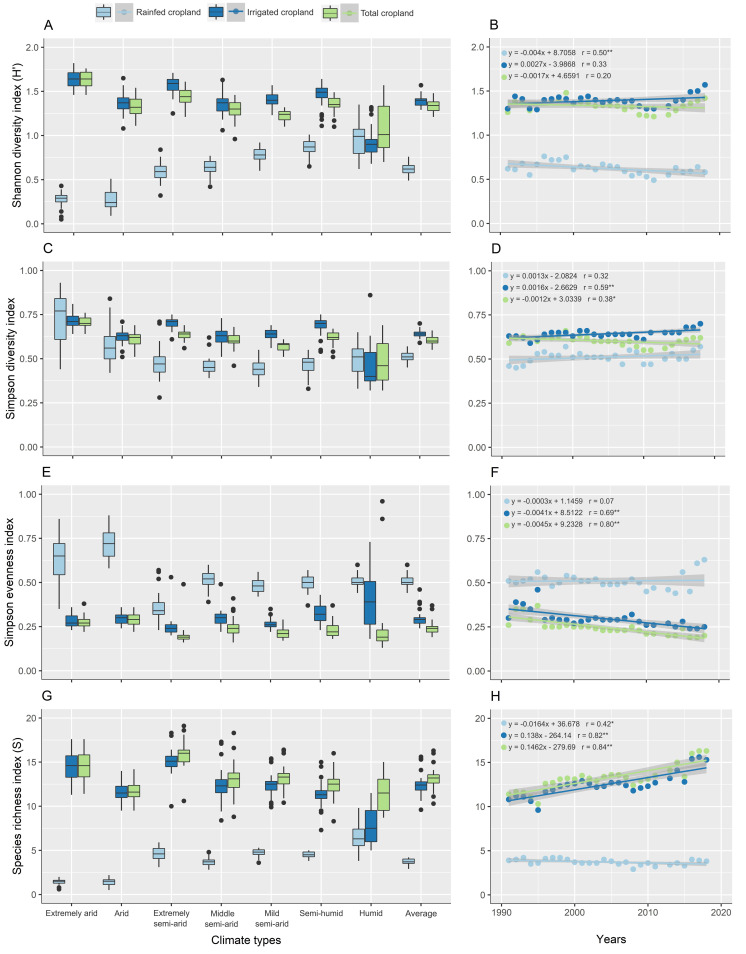
The box-plot of Shannon diversity index (H’) (A), Simpson diversity index (C), Simpson evenness index (E) and Species richness index (S) (G) in different climate and cultivation types of Iran, and the trend of the diversity (B, D) and evenness (F) indices and Species richness index (H) in different cultivation types of Iran between 1991 and 2018. The solid line is the linear regression fit to the points (n = 27) and 95% confidence interval shown by light-gray shading. Significant trends (p ≤ 0.01 and 0.05) shown by ** and *, respectively. Created using ggplot2 R-package.

The diversity indices were affected by the different climates of Iran. The trend of Shannon diversity index in rainfed cropland increased from extremely arid climate to humid climate in contrast to irrigated and total cropland. The average Shannon diversity index in rainfed, irrigated and total cropland in humid climate was almost equal. The highest and lowest Shannon diversity index were obtained in humid and extremely arid climates, respectively (0.96 and 0.27). In general, the average diversity of Shannon index was similar and higher in irrigated and total cropland in different climates than rainfed cropland (Fig 10A). Considering the Shannon value during 1991–2018, the trend of crop diversity was almost constant in irrigated and total cropland, while the Shannon diversity index in rainfed cropland showed a significant negative trend (r = 0.50** and slope = -0.002) (Fig 10B). Also, the change-points in the trend of Shannon index were observed in 1999 and 2010. The Shannon index had a decreasing trend between 1999 and 2010. After that, an increasing trend started until 2018 (Fig 10B).

Simpson diversity index was higher in extremely arid and humid climates in rainfed cropland than in irrigated and total cropland (Fig 10C). By reducing climatic aridity (from extremely arid climate to semi-humid climate), the amount of Simpson diversity index was lower in rainfed cropland than in irrigated and total cropland. But the average of Simpson diversity in irrigated and total cropland in different climates was similar and higher than in rainfed cropland (Fig 10C). On the other hand, the Simpson diversity index had an increasing trend in irrigated (p<0.01) and rainfed (p>0.01) cropland during 1991–2018 (Fig 10D), while this index in total cropland had a significant decreasing trend with a slope of -0.001 per year. The change-points in the trend of Simpson diversity index were almost similar to the Shannon index (Fig 10D).

In different climates, the Simpson evenness index was almost the same in irrigated and total cropland (0.32 and 0.25, respectively) and lower than in rainfed cropland (0.53) (Fig 10E). The highest difference between the evenness of crop species in irrigated and total cropland with rainfed cropland was observed in extremely arid and arid climates. By reducing climatic aridity, the evenness of crops in rainfed cropland also decreased, but it was still higher than in irrigated and total cropland. On average, the highest evenness of crop species was obtained in humid climate (0.47) due to increased evenness in irrigated cropland and arid (0.44) and extremely arid (0.40) climates due to increased evenness in rainfed cropland. In addition, the semi-arid climate had the lowest crop species evenness (0.27) (Fig 10E). In last three decades, the Simpson evenness index trend had a significant decrease in irrigated (r = 0.69** and slope = -0.004 year^-1^) and total (r = 0.80** and slope = -0.005 year^-1^) cropland (Fig 10F).

The crop species richness index in irrigated and total cropland was similar and higher than rainfed cropland in different climates (Fig 10G). By reducing climatic aridity (from extremely arid climate to semi-humid climate), the trend of species richness index in rainfed cropland was increased in contrast to irrigated and total cropland. In humid climate, the highest and lowest species richness index was observed in rainfed (6.62) and irrigated (8.07) cropland, which resulted in a decrease in species richness index in total cropland (8.68) (Fig 10G). Moreover, the species richness index had a significant positive trend in irrigated (r = 0.82** and slope = 0.14 year^-1^) and total cropland (r = 0.84** and slope = 0.15 year^-1^) in contrast to rainfed cropland (r = 0.42* and Slope = -0.02 year^-1^) during the last three decades (Fig 10H).

## Discussion

The diversity and evenness of crops are associated with and affected by numerous anthropogenic and natural factors in a particular place and time. Climate is one of the factors affecting and determining biodiversity in many parts of the world. In Iran, according to the study, crop diversity was affected by climate in such a way that crop diversity in rainfed cropland was much lower than in irrigated cropland due to lack of water for the cultivation of many crops. Shannon diversity index under rainfed cultivation, in contrast to irrigated and total croplands, dropped with increasing climatic aridity (from semi-humid climate to extremely arid climate). Also, the evenness of crops in rainfed cropland decreased with increasing climatic aridity (from extremely arid climate to semi-humid climate), but was still higher than in irrigated and total cropland. On average, the highest evenness of crop species was obtained in humid climate due to increased evenness in irrigated cropland and in arid and extremely arid climates due to increased evenness in rainfed cropland. Due to low average annual precipitation and the non-uniformity of annual precipitation distribution in Iran [2, 73], only crops such as wheat, barley and chickpea are grown in rainfed cropland, while in irrigated cropland, there is more crop diversity and crop species richness. Generally, the average annual precipitation in Iran was low except in the northern regions, which significantly impacted diversity and crop species richness of rainfed crops. Adler and Levine [74] also reported that changes in precipitation regimes under climate change are likely to affect species richness, especially in arid and semi-arid plant communities where water is the main limiting source. On the other hand, Jonas et al. [75] stated that the effect of climate on plant richness and diversity varies according to location and management practices such as fire, grazing and fertilization. As shown, water-limited regions such as Iran (with temporally and geographically uneven distribution) are most affected by climate change and its impacts (such as precipitation and temperature fluctuations) on the output of the agricultural sector [74, 76–78]. The climate in Iran is 35.5% very arid, 29.3% arid, 20.2% semi-arid, 5% Mediterranean and 10% humid [79]. Therefore, about 85% of Iranian cropland face significant water shortages and frequent droughts and are highly dependent on groundwater resources. In most regions, annual precipitation is less than 100 mm [80]. Over the last three decades, the precipitation in humid, semi-arid and arid regions has decreased by 59, 90 and 33 mm on average, respectively, and Tmax has increased by 0.7, 1.2 and 0.2°C, respectively [74]. In general, extreme weather events and droughts have potentially exacerbated Iran’s water problems by limiting the supply of renewable water and changing the spatial and temporal characteristics of temperature and rainfall [34, 76, 77]. In addition, most countries were affected by severe droughts in the late 1990s and 2008 [81, 82]. Finally, all the above issues affected the diversity and evenness of crops in Iran.

As shown, there is a significant relationship among biodiversity indices which are affected by the number and abundance of crop species. The relationship between indices was such that the value of Shannon index increased with increasing Simpson index and species richness. Specifically, species richness was associated with the evenness of crop species. The increasing number of crop species alone cannot improve crop diversity in a region because this increase may be associated with the dominance of one or more crop species. Only if crops cover a comparable area, crop diversity in the region increases. Although many crop species are cultivated in Iran, it does not significantly affect the calculated diversity indices due to their low area under cultivation. Koocheki et al. [83] reported a positive and significant correlation between Shannon diversity and species evenness indices. Their results also showed that the maximum and minimum values of Shannon index for the provinces of Iran were 1.17 and 0.36, which is consistent with the present study results.

Although the Shannon and Simpson indices have been subject to slight fluctuations in the past three decades, they remained more or less constant over time, while species richness increased. The increase in species richness is related to the recent increase in cultivated areas of particular crops. These indices have been fluctuating for several years for various reasons. Climate change (drought and temperature variability) and macro-agricultural policies (crop self-sufficiency, input subsidies, imports and exports, exchange rates, food security, and sanctions) have affected the diversity of Iranian crops by influencing the area under cultivation of crops.

Investigations showed that the cultivated area of wheat and barley had a significant impact on the diversity indices. Shannon diversity index was decreased when the dominance of wheat and barley increased per year due to a significant increase between their area under cultivation compared to other crop species of the country. Dominance is inversely related to diversity and causes instability and vulnerability of ecosystems. Hence, by increasing the area of wheat and barley and the prevalence of monoculture in each region, the probability of damages increased due to external factors such as sudden weather changes or the spread of diseases. Kiani et al. [84] reported that cereals were the dominant crops in Iranian agricultural production accounting for 45% of the cultivated area during 1981–2013. The cultivated area of wheat was increased by 80% and production by 347% in the last 50 years. In recent decades, the tendency to cultivate wheat in Iran has increased following the authorities’ emphasis on self-sufficiency in strategic cropping. Increasing government support policies to the agricultural sector, including guaranteed purchase, subsidies for pesticides and fertilizers, increased the area of wheat in the country, which led to the neglecting of some crops in Iran [85]. Similarly, In India, crop diversity declined sharply over a period of 60 years in certain regions, especially in rice and wheat production regions under the influence of the green revolution [86]. Government policies during the same period, including import subsidies, price controls and investment in the grain distribution network, may have helped increase the competitiveness of wheat and rice prices over other crops [87, 88]. Karbasi and Falsafizadeh [89] investigated the factors affecting crop diversity in Iran and reported a positive relationship between price, incentive policies (loans) and crop diversity. Koochaki et al. [90] reported that Iranian cropping systems are largely dominated by wheat and rice. While wheat is cultivated in most parts of Iran and rice is cultivated mostly on fringes of the Caspian Sea. They also pointed out that the highest area of cultivation in Iran is related to winter cereals. The reason for the development of cultivation of these crops is to adapt to environmental stress and low production costs.

On the other side, impressive pressure on surface and groundwater resources, as well as recent droughts, have led to the drying up of rivers, lakes, wetlands, aqueducts and springs, declining groundwater levels, draining of wells, soil erosion, desertification and frequent dust storms, loss of biodiversity and increasing pressure on rural livelihoods [91, 92], and eventually decline in food security in the future of Iran.

## Conclusions

Analyzing the statistical data allowed us to have a spatial and temporal estimate of crop diversity in different regions of Iran. The crop diversity in Iran was almost constant at national level during 1991–2018, but different trends were observed at sub-national level. These different trends in each region are influenced by various internal and external factors that affect the selection and cultivation of crops. The cultivation of wheat and barley in parts of Iran has reduced crop diversity in these regions, and on the other hand, these products are very important for the country in terms of food security. In general, the Iranian agricultural system is based on wheat cultivation which is the main crop in most crop rotations. Additionally, a significant difference was found between crop diversity in rainfed and irrigated cropland, which is affected by climatic conditions prevailing in Iran. The arid and semi-arid climate determines the cultivation of rainfed crops, and restricts farmers to cultivate a certain selection of plants. Finally, the study results indicated a low diversity of crops and an almost constant trend of crop diversity in agroecosystems of Iran, that their important consequences can be instability and increase the risk of production in the future.

## Supporting information

S1 Fig The ratio of cropland area of the studied sites to the total area of the sites (percentage) (bar plot) and the cropland area and total area of sites (thousand hectares) (text on a bar). (TIF)

S2 Fig Shannon diversity index (H’) trend of studied sites (67 sites) in total (A), rainfed (B) and irrigated cropland (C) in Iran between 1991 and 2018. Plots with no data also indicates the lack of rainfed cropland (B) at that site. Created using ggplot2 R-package.
(TIF)

S3 Fig Simpson diversity index trend of studied sites (67 sites) in total (A), rainfed (B) and irrigated cropland (C) in Iran between 1991 and 2018. Plots with no data also indicates the lack of rainfed cropland (B) at that site. Created using ggplot2 R-package.
(TIF)

S4 Fig Simpson evenness index trend of studied sites (67 sites) in total (A), rainfed (B) and irrigated cropland (C) in Iran between 1991 and 2018. Plots with no data also indicates the lack of rainfed cropland (B) at that site. Created using ggplot2 R-package.
(TIF)

S5 Fig Species richness index (S) trend of studied sites (67 sites) in total (A), rainfed (B) and irrigated cropland (C) in Iran between 1991 and 2018. Plots with no data also indicates the lack of rainfed cropland (B) at that site. Created using ggplot2 R-package.
(TIF)
