# Supplementary figures and images for: Temporal and regional shifts of crop species diversity in rainfed and irrigated cropland in Iran
Source: PLoS One. 2022 Mar 11;17(3):e0264702. doi: 10.1371/journal.pone.0264702 (PMC8947817; doi:10.1371/journal.pone.0264702)

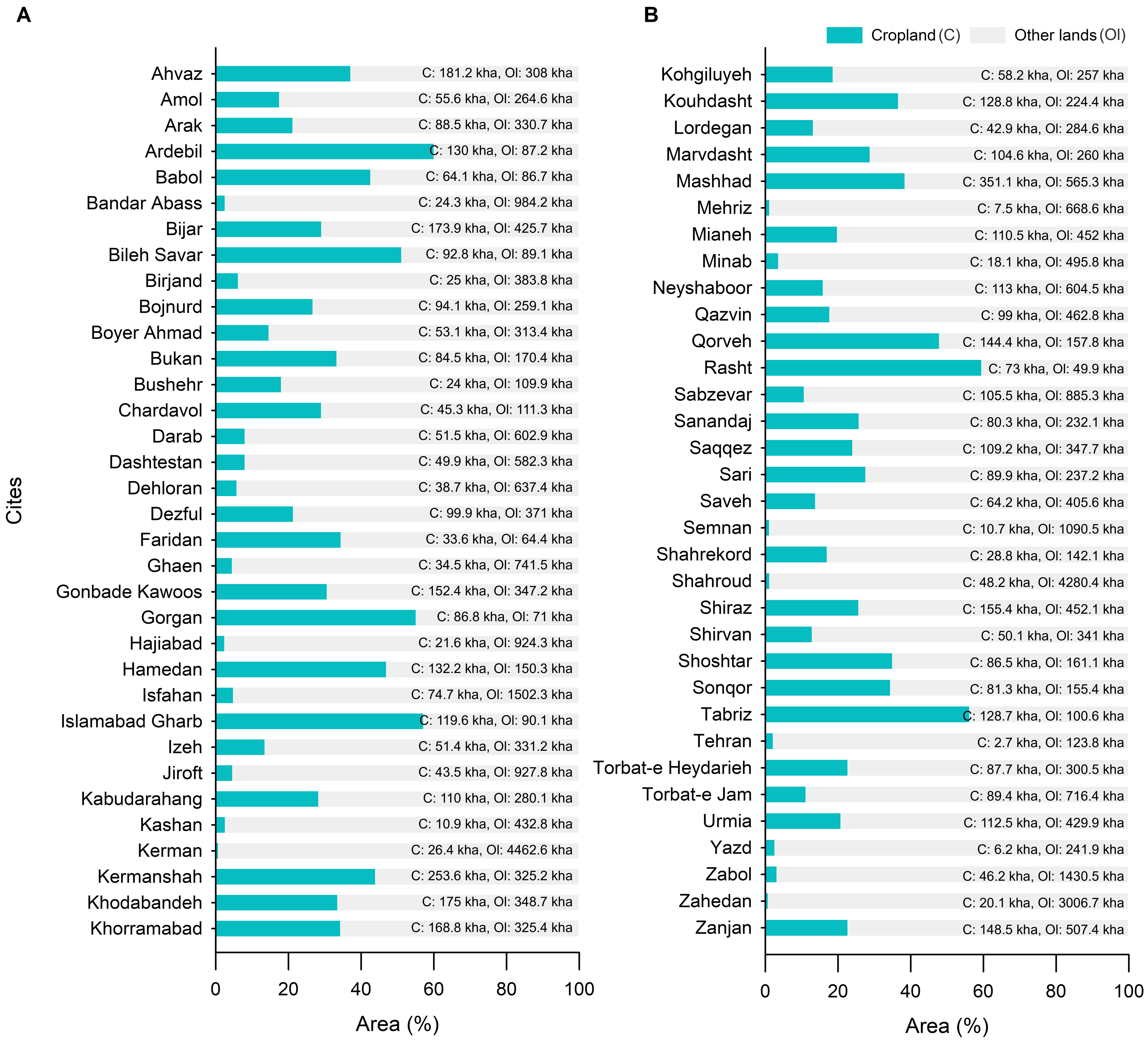

Supplement: S1 Fig — (TIF) [file pone.0264702.s001.tif]
